# Whole-Genome Sequencing Reveals Lignin-Degrading Capacity of a Ligninolytic Bacterium (*Bacillus cereus*) from Buffalo (*Bubalus bubalis*) Rumen

**DOI:** 10.3390/genes13050842

**Published:** 2022-05-08

**Authors:** Huimin Zhong, Jiayan Zhou, Fan Wang, Wenqing Wu, Mohamed Abdelrahman, Xiang Li

**Affiliations:** 1 National Center for International Research on Animal Genetics, Breeding and Reproduction (NCIRAGBR), Huazhong Agricultural University, Wuhan 430070, China; huiminzhong@webmail.hzau.edu.cn (H.Z.); jiayanzhou@webmail.hzau.edu.cn (J.Z.); wangfan6827@webmail.hzau.edu.cn (F.W.); wuwenqing@webmail.hzau.edu.cn (W.W.); 2 Key Laboratory of Agricultural Animal Genetics, Breeding and Reproduction of Ministry of Education, College of Animal Science and Technology, Huazhong Agricultural University, Wuhan 430070, China; mohamed.asad@agr.au.edu.eg; 3 Animal Production Department, Faculty of Agriculture, Assuit University, Asyut 71515, Egypt; 4 Shennongjia Science & Technology Innovation Center, Huazhong Agricultural University, Wuhan 430070, China

**Keywords:** *Bacillus cereus*, lignin-degrading, buffalo rumen, whole-genome sequencing, enzymology

## Abstract

The buffalo is an amazing ruminant. Its ability to degrade lignin, which has been recently reported, is most likely due to unique rumen microorganisms with lignin-degradation potential. Our goal was to explore the lignin-degradation potential of ruminal microorganisms, in which ligninolytic enzyme encoding genes were involved to provide ideas for revealing the mechanism of lignin degradation by buffalo. In this study, a bacterium strain identified as *Bacillus cereus* AH7-7 was isolated from the buffalo (*Bubalus bubalis*) rumen. After whole-genome sequencing, the results demonstrated that *B. cereus* AH7-7 had laccase, cytochrome P450 and vanillin alcohol oxidase-encoding genes. Sixty-four genes of *B. cereus* AH7-7 were involved in multiple aromatic metabolic pathways, such as phenylalanine metabolism and aminobenzoate degradation. A positive reaction resulting in guaiacol medium indicated that laccase secretion from *B. cereus* AH7-7 increased with time. A biodegradation experiment revealed that a significant reduction in kraft lignin content (25.9%) by *B. cereus* AH7-7 occurred at the end of 6 days of incubation, which confirmed its lignin-degradation capacity. Overall, this is the first report showing that *B. cereus* AH7-7 from the buffalo rumen can degrade lignin, and revealing the encoding genes of lignin-degrading enzymes from genome level.

## 1. Introduction

Lignin is an extremely stable aromatic three-dimensional polymer embedded between cellulose and hemicellulose in plants. It can reduce the nutritional value and utilisation of roughage and hinder the digestive ability of common animals [1]. Thus, the microbial degradation of lignin has become a research hotspot. At present, it has been shown that proteobacteria and actinomycetes are the main bacteria phyla with lignin-degradation functions [2,3]. Bacteria such as *Sphingomonas* [4], *Pseudomonas* [5], *Rhodococcus* [6], *Nocardia* [7,8] and *Bacillus* [9] possess the capacity to degrade lignin; however, the genomic characteristics of most lignin-degrading bacteria at the whole genome level still remain unknown. The typical lignin-degrading enzymes laccase [10] and dye decolourising peroxidase [11] are widespread in bacteria, and perform the function of degrading lignin. Moreover, bacteria contain another set of lignin-degrading enzymes, including monooxygenases such as cytochrome P450 [12], dioxygenase [13], phenoloxidase [14], catalase-peroxidase [11], glutathione s-transferase [4,15,16], glutathione dependence-β-etherase [17], glutathione lyase [18] and manganese-dependent superoxide dismutase [19]. Gene level research can better reveal the ability of a strain to produce lignin degrading enzymes. For example, research reported that the structural difference in the 5′UTR region of laccase type genes could influence the expression of the genes [20]. Transformation at the gene level is also a current research direction, the overexpression of a laccase-encoding gene providing *G. trabeum* with ligninolytic activity on wood [21].

Buffalos have a strong tolerance to roughage, and were reported as the first mammal to degrade lignin efficiently [22]. Studies found that the digestibility of crude protein, dry matter and organic matter in rice straw roughage in the buffalo rumen was significantly higher than in cattle, and the utilisation of acid detergent fibre was also more efficient [23,24]. The peculiar rumen microbial system of buffalos is the reason why the buffalo grows more than cattle in a long dry season without green grass. This ability for lignin degradation might be due to the unique rumen microflora of buffalo. Many studies have shown that the amount of the cellulolytic bacteria *Ruminococcus albus* in buffalo rumen is significantly higher than in beef cattle, and the total number of cellulolytic, proteolytic and amylolytic bacteria of buffalo rumen fed with rice straw was significantly higher than in cattle fed the same way [24]. Wang et al. isolated three strains with lignin-degradation potential from the buffalo rumen for the first time: *Ochrobactrum pseudintermedium*, *Klebsiella pneumoniae* and *Bacillus sonorensis* [25]. Similarly, six strains with lignin degradation potential have been recently isolated [26].

We hypothesised that the ability of buffalo ruminal lignin-degrading bacteria results from its unique ligninolytic enzymatic system. The present study aimed to explore the lignin-degrading ability of potentially ligninolytic strains based on genomics and enzymology. Our findings may shed light on the mechanism of lignin degradation by buffalo.

## 2. Materials and Methods

### 2.1. Isolation and Identification of Lignin-Degrading Bacteria

As inoculum material, rumen content collected from fistula buffalos raised in Hubei Jingniu Animal Husbandry Co., Ltd was used. To avoid the disturbance from forage, the rumen content was collected from three fistula buffalos (Mediterranean × Nili-Ravi, seven years old, 572 ± 24 kg) after 24 h of fasting. Enriched cultures from enriched medium were transferred to the lignin-degrading bacteria screening medium and grown under anaerobic conditions at 39 °C. Over a 10-day period, three consecutive transfers were performed and then the cultures were streaked onto Luria Broth agar to obtain pure cultures.

The compositions of the media were as follows:

Rumen fluid: the rumen content was filtered through four layers of clean gauze to obtain the supernatant fluid, the supernatant fluid from three buffalos was mixed in equal proportion, and then the rumen fluid centrifuged at 9000 rpm at 4 °C for 10 min.

Phosphate-buffered mineral salts medium A (per litre): [(NH_4_)_2_SO_4_, 3.0 g; NaCl, 6.0 g; KH_2_PO_4_, 3.0 g; CaCl_2_·2H_2_O, 0.4 g; MgSO_4_·7H_2_O, 0.6 g.

Phosphate-buffered mineral salts medium B (per litre): K_2_HPO_4_·3H_2_O, 4.0 g.

Enrichment medium (per litre): 170 mL rumen fluid, 165 mL phosphate-buffered mineral salts medium A, 165 mL phosphate-buffered mineral salts medium B, 0.5 mg copper sulphate, 1.0 g tryptone, 1.0 g yeast extract.

Screening medium (per litre): 165 mL phosphate-buffered mineral salts medium A, 165 mL phosphate-buffered mineral salts medium B, 5.0 g sodium lignosulfonate.

The bacterial strain eventually isolated from the buffalo rumen was named AH7-7. A purified strain of AH7-7 was successively diluted with sterile saline, streaked onto the Luria Broth agar, incubated at 39 °C for 24 h, and then the characteristics of colonies were observed. The isolated strain AH7-7 was observed by Gram staining. Classical physiological and biochemical characteristics of strain AH7-7 were tentatively identified according to the Taxonomic Outline of the Prokaryotes Bergey’s Manual of Systematic Bacteriology [27].

Bacterial 16S rRNA sequencing and gyrB sequencing were used for molecular identification. For PCR amplification in 16S rRNA sequencing, 27F (5′-GAGTTTGATCCTGGCTCAG-3′) was used as the forward primer and 1492R (5′-TACGGTTACCTTGTTACGACTT-3′) as the reverse primer. For gyrB sequencing, gyrbF (5′-ATTGGTGACACCGATCAAACA-3′) was used as the forward primer and gyrbR (5′-TCATACGTATGGATGTTATTC-3′) as the reverse primer. PCR amplification was at a total reaction volume of 30 µL, which included 10.5 µL nuclease free water, 1.5 µL of each primer, 1.5 µL genomic DNA and 15 µL Master Mix (KOD ONE MM, TOYOBO). For the PCR setup, the initial denaturation step was performed at 95 °C for 2 min, followed by 25 cycles (denaturation at 98 °C for 10 s, program of annealing at 55 °C for 30 s, extension at 72 °C for 90 s), and the final extension step was performed at 72 °C for 2 min. Bacterial 16S rRNA sequencing and gyrB sequencing were performed by the China Centre for Type Culture Collection. The sequencing results were compared with the data on the NCBI database (accessed on 30 November 2020). Drawing of a phylogenetic tree was completed by MEGA7 software with the statistical method of maximun likelihood. The bootstrap method was used for test of phylogeny. The number of bootstrap replications was 1000.

### 2.2. DNA Library Preparation, Whole-Genome Sequencing and Assembly

The enrichment medium of lignin-degrading bacteria was used for pure culture of strain AH7-7. The CTAB method was adopted to extract DNA, and the total amount of DNA was detected by a Quant-IT Pico-Green dsDNA assay kit (Invitrogen, Shanghai, China). Second-generation libraries were made according to the Illumina TruSeq Nano DNA LT library preparation process. The experimental process was based on a TruSeq™ DNA Sample Prep kit. Next-generation sequencing libraries were sequenced based on the Illumina NovaSeq sequencing platform, paired-end 2 × 150 bp sequencing mode, and the library insert size was 400 bp. The steps of third-generation library preparation followed the protocol formulated by Oxford Nanopore Technologies, including sample quality inspection, library construction, library-quality inspection and library sequencing. Nanodrop, Qubit and 0.35% agarose gel electrophoresis were used to conduct a quality inspection of genomic DNA. An automatic nucleic acid recovery machine (BluePippin) was used to recover large-fragment DNA. To build the library, we used an SQK-LSK109 ligation kit (Oxford Nanopore Technologies, Oxford, UK). The third-generation libraries were sequenced based on the Oxford Nanopore ONT sequencing platform.

FastQC0.11.8 was used to control the quality of the next-generation sequencing data [28], and AdapterRemoval2.2.2 was used to remove contaminated low-quality reads from the 3′ end connector [29]. SOAPdenovo2 was used to calibrate the quality of all reads based on the Kmer frequency; the Kmer was set to 17 [30]. Three generations of data were assembled by HGAP3 [31] and CANU1.8 [20] to obtain the contig sequences. Pilon1.23 was used to correct the next-generation high-quality data for the third-generation contig results [32]; the complete sequence was eventually obtained. Personabio Biotechnology Co., Ltd. (Shanghai, China) completed the whole-genome sequencing and assembly procedures.

### 2.3. Open Reading Frames (ORFs), Evolutionary Genealogy of Genes: Non-Supervised Orthologous Groups (eggNOG), Kyoto Encyclopedia of Genes and Genomes (KEGG) and Carbohydrate-Active Enzymes Analysis

The software used for ORFs prediction was GeneMarkS (version v4.32, http://topaz.gatech.edu/GeneMark/, accessed on 10 December 2020) [33]. All of the ORFs obtained by whole gene sequencing were annotated through eggNOG (version v20171128, http://eggnogdb.embl.de/#/app/home/, accessed on 10 December 2020) [34] and the online version of the KEGG database (http://www.genome.jp/kegg/, accessed on 16 December 2020) [35]. CAZy software (version v6, http://www.cazy.org/, accessed on 16 December 2020) for carbohydrate-active enzymes prediction [36].

### 2.4. Guaiacol Colour Reaction

A single colony of AH7-7 was selected and inoculated in laccase qualitative medium (guaiacol medium) placed under aerobic conditions at 39 °C in the incubator. The colour change of the medium was observed at different times. When guaiacol reacts with laccase, a positive reaction appears, showing a reddish-brown halo. Guaiacol medium (per litre) contained 10.00 g NaCl, 10.00 g tryptone, 5.00 g yeast powder, 20.00 g agar and 0.04% guaiacol. Inoculating in medium that had no guaiacol was used as control.

### 2.5. Biodegradation of Kraft Lignin by Strain B. cereus AH7-7

A biodegradation experiment was conducted in a 250 mL flask with 100 mL sterile kraft lignin containing mineral salt medium, pH at 6.5. The concentration of kraft lignin in the degradation medium was 1000 mg/L. Bacterial culture liquid with 10^6^ CFU/mL was inoculated into triplicate flasks; each flask was inoculated 1 mL. Uninoculated medium was used as a control. The flasks were incubated on a rotary shaker under aerobic conditions at 39 °C and 120 rpm for 6 days. Samples were extracted periodically once a day and the content of kraft lignin determined. After centrifugation at 8000× *g* for 10 min, the centrifuged supernatants of the inoculation and control groups were acidified to pH 1–2 with 12 M HCl, then the precipitate was collected by centrifugation at 12,000× *g* for 10 min. Each precipitate was washed with deionized water, dried at 65 °C for 48 h and weighed to obtain residual kraft lignin [37].

## 3. Results

### 3.1. Identification of Strain AH7-7

**Figure 1:**
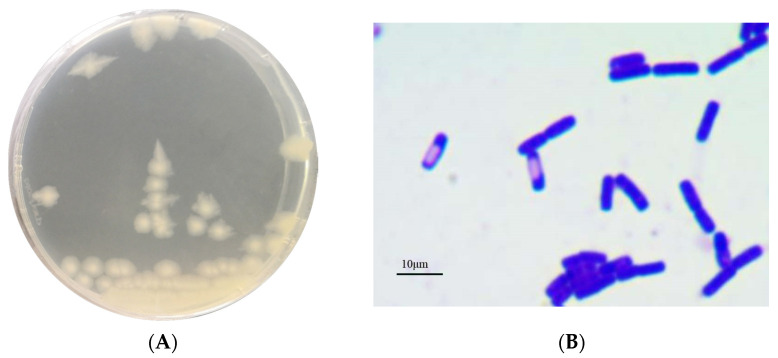
(**A**) Observation of colonies from strain AH7-7. (**B**) Gram staining of strain AH7-7 under the microscope (magnification 1000×, Olympus BX53).

**Figure 2:**
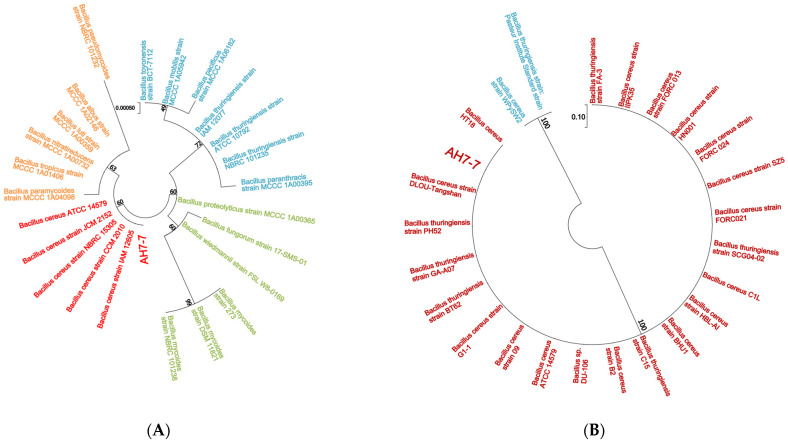
Phylogenetic tree of strain AH7-7. (**A**) 16S rRNA; (**B**) gyrB.

The colonies were large, waxy, round, translucent and greyish white, with rough surfaces and irregular edges (Figure 1A). The strain was Gram-positive according to Gram staining observed under the microscope (Figure 1B). The phylogenetic tree of the 16S rRNA gene and gyrB gene indicated that strain AH7-7 was the closest to *B. cereus* (Figure 2). Detail of blast analysis of the 16S rRNA gene and gyrB gene are shown in Table A1 and Table A2. The results of physiological and biochemical characteristics of strain AH7-7 are shown in Table A3 and Table A4. According to colony observation, Gram staining, physiological and biochemical data, 16S rRNA sequencing and gyrB gene sequencing, strain AH7-7 was identified as *B. cereus* AH7-7.

### 3.2. Basic Genomic Characteristics of B. cereus AH7-7

**Figure 3:**
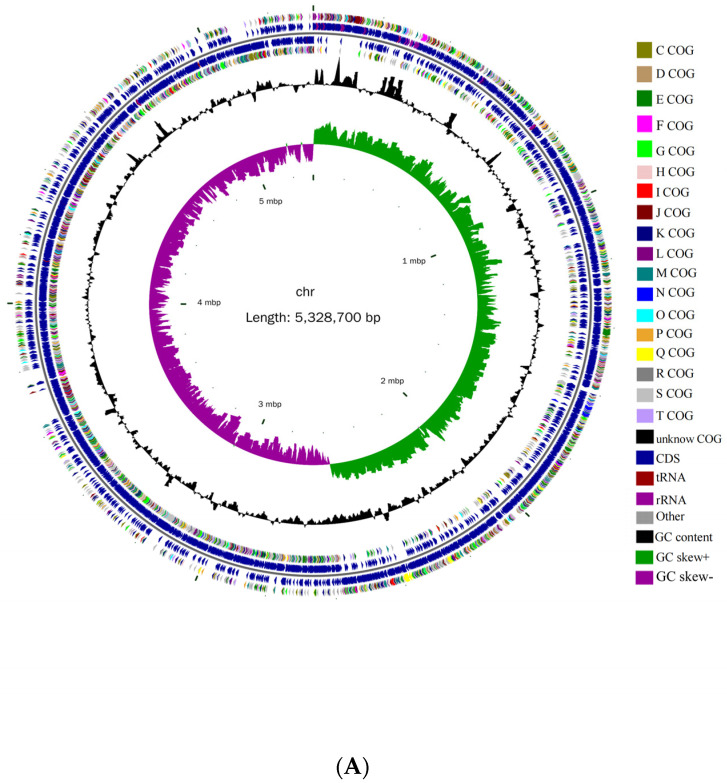

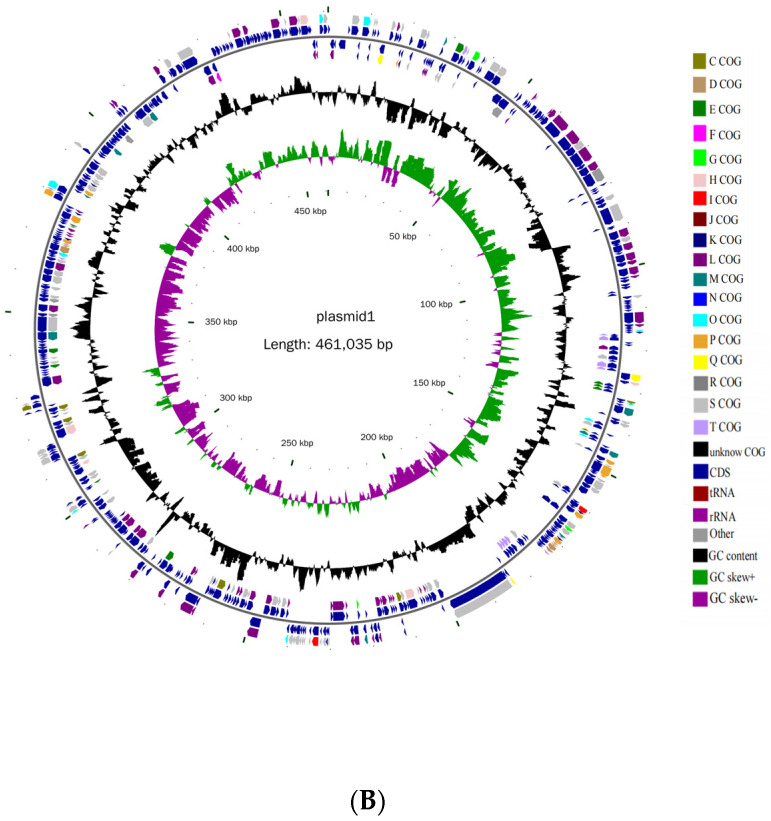
Genomic architecture of *B. cereus* AH7-7. (**A**) Genome map and (**B**) plasmid map. Each circle, from centre to the outside, represents the following features. The first circle represents the scale mark, the second circle represents GC skew, the third circle represents GC content, the fourth and seventh circles represent every COG to which each coding sequence (CDS) belongs, and the fifth and sixth circles represent the locations of CDS, tRNA and rRNA in the genome.

Whole-genome sequencing results indicated that *B. cereus* AH7-7 consists of a chromosome and a circular plasmid of 5,328,700 bp and 461,035 bp, respectively; the GC content is 35.36% and 33.67%, respectively (Table 1). There are 5440 ORFs in the chromosome and 455 ORFs in the plasmid. The genome circle map of *B. cereus* AH7-7 is shown in Figure 3.

### 3.3. Annotation Results of B. cereus AH7-7 in eggNOG, KEGG and Carbohydrate-Active Enzymes Database

In the eggNOG database, in the chromosome genome, one ORF was annotated as a laccase gene, one ORF as multicopper oxidase gene, five ORF as cytochrome P450 genes, eight ORFs as monooxygenase genes, 30 ORFs as dioxygenase genes, seven ORFs as catalase genes, 36 ORFs as oxidase genes, 28 ORFs as oxidoreductase genes, and 109 ORFs as dehydrogenase genes. In the plasmid genome, the results showed that one ORF was annotated as multicopper oxidase gene, one ORF as cytochrome P450 gene, one ORF as oxidoreductase gene, six ORFs as dehydrogenases genes (Table 2).

**Figure 4:**
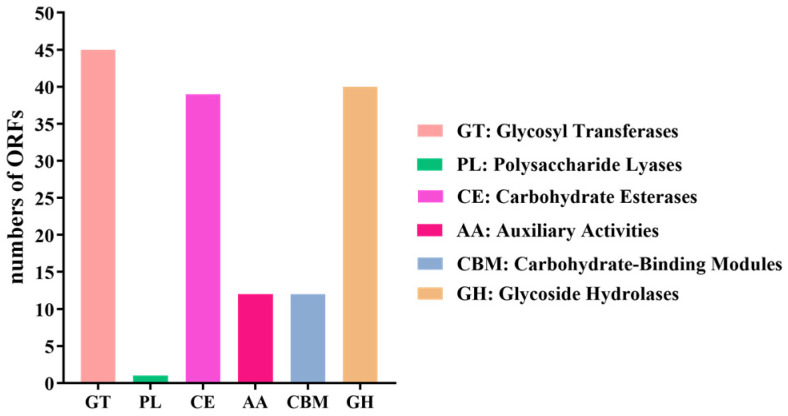
Annotation of *B. cereus* AH7-7 carbohydrate-active enzymes.

In the carbohydrate-active enzymes database, a total 45 ORFs were annotated as glycosyl transferases genes, one ORF as a polysaccharide lyase gene, 39 ORFs as carbohydrate esterase genes, 12 ORFs as auxiliary activities genes, 12 ORFs as carbohydrate-binding modules genes and 40 ORFs as glycoside hydrolases genes (Figure 4). As shown in Table 3, two ORFs were annotated as vanillin alcohol oxidase genes, two ORFs as 1,4-benzoquinone reductase genes, three ORFs as glucooligosaccharide oxidase, chitooligosaccharide oxidase or cellooligosaccharide dehydrogenase genes, three ORFs as monooxygenase genes, and two ORFs as laccase or dihydrogeodin oxidase.

**Figure 5:**
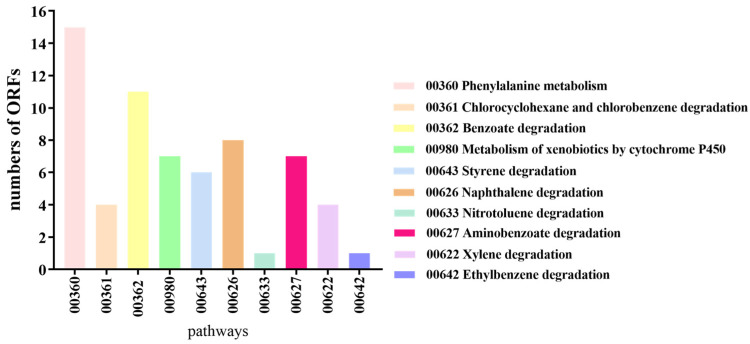
The number of genes of *B. cereus* AH7-7 enriched in KEGG pathways related to aromatic degradation.

In the KEGG database, 15 ORFs of *B. cereus* AH7-7 were linked to phenylalanine metabolism, four to chlorocyclohexane and chlorobenzene degradation, 11 to benzoate degradation, seven to the metabolism of xenobiotics by cytochrome P450, six to styrene degradation, eight to naphthalene degradation, one to nitrotoluene degradation, seven to aminobenzoate degradation, four to xylene degradation and one to ethylbenzene degradation (Figure 5).

### 3.4. Laccase Secretion of B. cereus AH7-7

**Figure 6:**
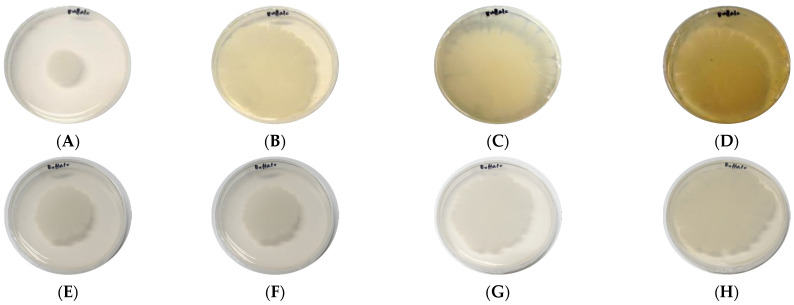
Colour reaction of guaiacol and kraft-lignin reduction. (**A**) guaiacol: 1 d. (**B**) guaiacol: 4 d. (**C**) guaiacol: 10 d. (**D**) guaiacol: 15 d. (**E**) control: 1 d. (**F**) control: 4 d. (**G**) control: 10 d. (**H**) control: 15 d.

The guaiacol medium determined the laccase secretion of *B. cereus* AH7-7. The strain began to show a slight reddish-brown halo from the 4th day after culture, deepened gradually on the 10th day and showed obvious colour development on the 15th day (Figure 6).

### 3.5. Kraft-Lignin Degradation of B. cereus AH7-7

**Figure 7:**
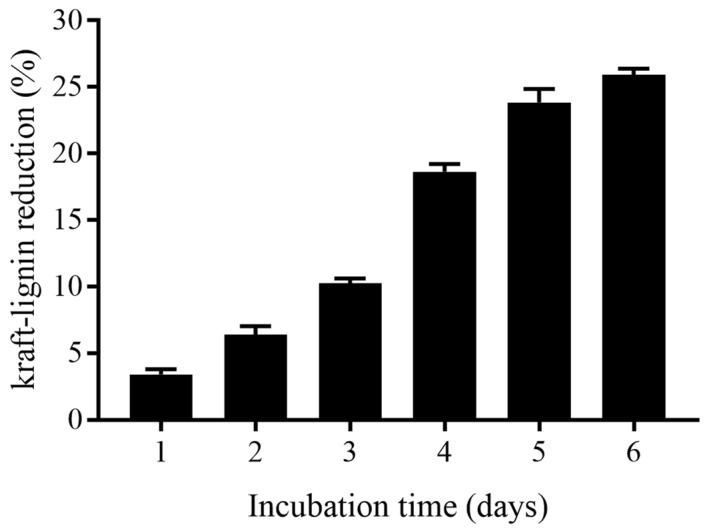
Kraft-lignin reduction.

The reduction in kraft-lignin content by the *B. cereus* AH7-7 degraded sample is shown in Figure 7. There was a minor reduction of kraft lignin content in the medium during the first two days of culture. A significant reduction in kraft lignin content (25.9%) was observed after 6 days of incubation.

## 4. Discussion

There are strong interactions between the rumen microbiome and buffalo host. Buffalos live in tropical areas with high temperatures and lack of high-quality feed. Buffalos are mainly fed low-quality roughage, such as rice straw and field crops [38]. Because of the intake of roughage with high fibre, some bacteria with lignin degradation ability have been naturally selected over time, so that buffalos have developed a unique rumen microflora in the long-term domestication process. In a previous study, buffalo lignin-degrading capacity was first demonstrated, and the buffalo was found to be the first mammal with the ability to degrade lignin [22]. Strains with the potential of degrading lignin were isolated from buffalo rumen [25]; however, Wang et al. did not carry out further studies. Our results verified previous results in that the ability of buffalo to degrade lignin was found to be intimately related to rumen microorganisms. *B. cereus* AH7-7 from the buffalo rumen made a significant reduction of kraft lignin content (25.9%) after 6 days of incubation. Therefore, it is important to understand how this animal degrades lignin via its rumen microbial system.

Laccase, first discovered in 1883 from *Rhus verniciflua,* is a typical lignin-degrading enzymes. A laccase molecule contains four copper ions to oxidise a series of aromatics [39]. Since 1993, when laccase was identified in *Azospirillum lipoferum* [40], many bacteria have been classified as laccase-secreting bacteria due to the development of technology [41,42,43]. This study annotated all ORFs of the *B. cereus* AH7-7 in the eggNOG database. We showed that an ORF was annotated as a laccase gene, and two ORFs were annotated as multicopper oxidase genes. The ORFs identified as multicopper oxidase genes were closely related to laccase activity in the carbohydrate-active enzymes database. Thus, *B. cereus* AH7-7 may have more than one gene that encodes laccase. Consistent with the above results, the colour reaction of guaiacol showed that *B. cereus* AH7-7 has the capacity for laccase secretion, and the amount increased with the increasing of time. Xu proposed that bacterial laccase had significant advantages over fungi with respect to high temperature, salt and pH resistance [44]. Laccase obtained from *Bacillus halodurans* had better thermal stability and more robust alkaline tolerance [45]. These results show that *B. cereus* AH7-7 can degrade lignin by secreting laccase, and more effectively than fungi in lignin degradation. Monooxygenase and dioxygenase have been also been shown to play an important role in lignin degradation. Previous research confirmed that cytochrome P450 belongs to a monooxygenase that involves lignin metabolism [12]. In the eggNOG database, five ORFs of *B. cereus* AH7-7 were annotated as the cytochrome P450 gene, coding the ligninolytic enzyme cytochrome P450 and may be involved in the lignin degradation process. However, lignin degradation is a complex process involving dehydrogenation and oxidation-reduction steps. These are probably catalysed by enzymes that were not found. In the eggNOG and the carbohydrate-active enzymes databases, large categories such as oxidase, oxidoreductase and dehydrogenase have been summarised, but we still do not know to which specific enzymes they relate.

According to the KEGG result, seven genes were annotated in the pathway of aminobenzoate degradation. A study indicated that the aminobenzoate degradation pathway includes two branches of lignin metabolism, named the multiple 3-O-methyl gallate catabolic pathway and the protocatechuate 4,5-cleavage pathway [46]. Vanillin is a vital intermediate in the two lignin biodegradation pathways. In this study, when the ORFs were annotated in the carbohydrate-active enzymes database, *B. cereus* AH7-7 was found to have genes that could encode vanillin alcohol oxidase. The vanillin alcohol oxidase may be the key factor involved in the lignin degradation by *B. cereus* AH7-7 via the above two pathways. Masai et al. have shown that in multiple 3-O-methyl gallate catabolic pathways, syringate could be catalysed by tetrahydrofolate-dependent demethylase to produce methyl gallate, which can be converted into 4-oxalomesaconate through a series of reactions [46]. Moreover, in the protocatechuate 4,5-cleavage pathway, vanillin was first catalysed to protocatechuate, and finally to oxaloacetic acid and pyruvic acid. Thus, two established lignin metabolic pathways may also be involved in the main process of lignin metabolism by *B. cereus* AH7-7.

## 5. Conclusions

The present study indicated that *B. cereus* AH7-7 isolated from the buffalo rumen content has the capacity for lignin degradation. *B. cereus* AH7-7 has laccase, cytochrome P450 and vanillin alcohol oxidase genes that can produce enzymes involved in lignin degradation. Furthermore, laccase secretion and capacity of kraft-lignin degradation by *B. cereus* AH7-7 increased with time.

## Figures and Tables

**Table 1:** Genomic characteristics of *B. cereus* AH7-7.

Genomic Contents	Chromosome	Plasmid
Sequence length (bp)	5,328,700	461,035
GC content (%)	35.36	33.67
Open reading frames	5440	455
Number of tRNAs	107	0
Number of 5rRNAs	14	0
Number of 6rRNAs	14	0
Number of 23rRNAs	14	0
Number of ncRNAs	321	12

**Table 2:** Enzymes that related to lignin degrading in functional annotation of eggNOG.

Enzymes	Chromosome	Gene IDs	Plasmid	Gene IDs
Laccase	1	chr_3833	0	-
Multicopper oxidase	1	chr_1866	1	plasmid1_118
Cytochrome P450	5	chr_2531, chr_2535	1	plasmid1_13
Monooxygenase	8	chr_1061, chr_1702	0	-
Dioxygenase	30	chr_243, chr_244	0	-
Catalase	7	chr_782, chr_823	0	-
Oxidase	36	chr_185, chr_397	0	-
Oxidoreductase	28	chr_280, chr_384	1	plasmid1_377
Dehydrogenase	109	chr_183, chr_325	6	plasmid1_193, plasmid1_241

Note: Only two IDs are listed in the table when more than two genes are found. “-” means none. Hit names of Gene IDs in NR are shown in Supplementary Table S1. Detail sequences information of Gene IDs are shown in Supplementary Material.

**Table 3:** Category and number of annotated open reading frames (ORFs) of auxiliary activities family in *B. cereus* AH7-7.

Auxiliary Activities	Number of ORFs Annotated	Genes IDs	Enzymes
AA1_3	2	chr_1866, plasmid1_118	laccase (EC 1.10.3.2); dihydrogeodin oxidase (EC 1.10.3)
AA4	2	chr_1325, chr_3451	vanillin alcohol oxidase (EC 1.1.3.38)
AA6	2	chr_1403, chr_5117	1,4-benzoquinone reductase (EC. 1.6.5.6)
AA7	3	chr_185, chr_397, chr_649	glucooligosaccharide oxidase (EC 1.1.3); chitooligosaccharide oxidase (EC 1.1.3); cellooligosaccharide dehydrogenase (EC 1.1.99)
AA10	3	chr_2698, chr_2731, plasmid1_37	monooxygenase (EC 1.14.99.54; EC 1.14.99.56; EC 1.14.99.53; EC 1.14.99)

Note: Hit names of Gene IDs in NR are shown in Supplementary Table S1. Detail sequences information of Gene IDs are shown in Supplementary Material.

## Data Availability

The original contributions presented in the study are included in the article/Supplementary Material. The datasets used and/or analysed during the current study are available from the corresponding author. All the sequences were submitted on NCBI. For WGS raw data, the accession numbers are SRR17994727 and SRR17994728, the bioproject accession is PRJNA806339, and the biosample accession is SAMN25872064. The accession of 16S rRNA gene of strain AH7-7 is OM993330. The accession of gyrB gene of strain AH7-7 is BankIt2562125 Sequence_AH7-7 ON000289.

## Supplementary Materials

The following supporting information can be downloaded at: https://www.mdpi.com/article/10.3390/genes13050842/s1, Table S1: Hit names of Gene IDs in NR.

## Appendix A

**Table A1:** Blast results of the 16S rRNA gene.

Query Acc.ver	Subject Acc.ver	% Identity	Alignment Length	Mismatches	Gap Opens	Q. Start	Q. End	S. Start	S. End	Evalue	Bit Score
AH7-7	NR_115526.1	99.86	1424	0	2	3	1424	29	1452	0	2617
AH7-7	NR_115714.1	99.86	1424	0	2	3	1424	49	1472	0	2617
AH7-7	NR_112630.1	99.86	1424	0	2	3	1424	28	1451	0	2617
AH7-7	NR_113266.1	99.86	1424	0	2	3	1424	29	1452	0	2617
AH7-7	NR_114582.1	99.86	1424	0	2	3	1424	39	1462	0	2617
AH7-7	NR_152692.1	99.789	1424	1	2	3	1424	49	1472	0	2612
AH7-7	NR_157736.1	99.789	1424	1	2	3	1424	49	1472	0	2612
AH7-7	NR_157735.1	99.789	1424	1	2	3	1424	49	1472	0	2612
AH7-7	NR_157732.1	99.789	1424	1	2	3	1424	49	1472	0	2612
AH7-7	NR_157730.1	99.789	1424	1	2	3	1424	49	1472	0	2612
AH7-7	NR_157729.1	99.789	1424	1	2	3	1424	49	1472	0	2612
AH7-7	NR_157734.1	99.719	1424	2	2	3	1424	49	1472	0	2606
AH7-7	NR_170494.1	99.719	1424	2	2	3	1424	80	1503	0	2606
AH7-7	NR_157733.1	99.649	1424	3	2	3	1424	49	1472	0	2601
AH7-7	NR_157728.1	99.649	1424	3	2	3	1424	49	1472	0	2601
AH7-7	NR_043403.1	99.579	1424	4	2	3	1424	29	1452	0	2595
AH7-7	NR_121761.1	99.579	1424	4	2	3	1424	46	1469	0	2595
AH7-7	NR_114581.1	99.579	1424	4	2	3	1424	39	1462	0	2595
AH7-7	NR_112780.1	99.508	1424	5	2	3	1424	29	1452	0	2591
AH7-7	NR_157731.1	99.508	1424	5	2	3	1424	49	1472	0	2590
AH7-7	NR_113991.1	99.438	1424	6	2	3	1424	29	1452	0	2584
AH7-7	NR_036880.1	99.368	1424	7	2	3	1424	25	1448	0	2579
AH7-7	NR_024697.1	99.368	1424	7	2	3	1424	43	1466	0	2579
AH7-7	NR_113996.1	99.368	1424	7	2	3	1424	29	1452	0	2579

**Table A2:** Blast results of the gyrB gene.

Query Acc.ver	Subject Acc.ver	% Identity	Alignment Length	Mismatches	Gap Opens	Q. Start	Q. End	S. Start	S. End	Evalue	Bit Score
AH7-7	CP053289.1	100	343	0	0	1	343	42,757	42,415	2.08 × 10^−177^	634
AH7-7	CP040340.1	100	343	0	0	1	343	621,370	621,712	2.08 × 10^−177^	634
AH7-7	KY283089.1	100	343	0	0	1	343	91	433	2.08 × 10^−177^	634
AH7-7	CP042270.1	100	343	0	0	1	343	1,208,589	1,208,931	2.08 × 10^−177^	634
AH7-7	CP044978.1	100	343	0	0	1	343	791,108	791,450	2.08 × 10^−177^	634
AH7-7	CP042929.1	100	343	0	0	1	343	4959	5301	2.08 × 10^−177^	634
AH7-7	CP042874.1	100	343	0	0	1	343	4959	5301	2.08 × 10^−177^	634
AH7-7	CP034551.1	100	343	0	0	1	343	4677	5019	2.08 × 10^−177^	634
AH7-7	CP026607.1	100	343	0	0	1	343	4781	5123	2.08 × 10^−177^	634
AH7-7	MH427046.1	100	343	0	0	1	343	107	449	2.08 × 10^−177^	634
AH7-7	CP021436.1	100	343	0	0	1	343	5096	5438	2.08 × 10^−177^	634
AH7-7	CP023727.1	100	343	0	0	1	343	5,200,294	5,200,636	2.08 × 10^−177^	634
AH7-7	CP023245.1	100	343	0	0	1	343	4677	5019	2.08 × 10^−177^	634
AH7-7	CP022445.1	100	343	0	0	1	343	4677	5019	2.08 × 10^−177^	634
AH7-7	CP017577.1	100	343	0	0	1	343	4,203,994	4,204,336	2.08 × 10^−177^	634
AH7-7	CP014486.1	100	343	0	0	1	343	4967	5309	2.08 × 10^−177^	634
AH7-7	KT923660.1	100	343	0	0	1	343	107	449	2.08 × 10^−177^	634
AH7-7	CP012691.1	100	343	0	0	1	343	4677	5019	2.08 × 10^−177^	634
AH7-7	CP011155.1	100	343	0	0	1	343	5083	5425	2.08 × 10^−177^	634
AH7-7	CP011145.1	100	343	0	0	1	343	4677	5019	2.08 × 10^−177^	634
AH7-7	KJ813006.1	100	343	0	0	1	343	88	430	2.08 × 10^−177^	634
AH7-7	AP014864.1	100	343	0	0	1	343	5,891,887	5,891,545	2.08 × 10^−177^	634
AH7-7	CP068984.1	100	343	0	0	1	343	5470	5812	2.08 × 10^−177^	634
AH7-7	AP024504.2	100	343	0	0	1	343	4677	5019	2.08 × 10^−177^	634

**Table A3:** Physiological and biochemical characteristics of strain AH7-7 in the API20E identification system.

Substrate	Fermentation/Oxidation	Enzyme Activity	Results	Substrate Utilisation	Results
Glucose	−	β-Galactosidase	−	Citrate sodium	+
Mannitol	−	Arginine bishydrolase	+	Sodium thiosulfate (production of H2S)	−
Inose	−	Lysine decarboxylase	−	Tryptophan (production of indole)	−
Sorbic alcohol	−	Ornithine decarboxylase	−	Pyruvate (production of acetylmethyl methanol)	w
Rhamnose	−	Urease	−		
Saccharose	−	Tryptophan deaminase	−		
Melibiose	−	Gelatinase	+		
Amygdalin	−				
Arabinose	−				

Note: + means positive reaction, − means negative reaction, w means weakly positive reaction.

**Table A4:** Physiological and biochemical characteristics of strain AH7-7 in the API50CH identification system.

Substrate	Results	Substrate	Results	Substrate	Results
Control	−	Urosside	+	Glucose	+
Glycerol	−	Aesculin	+	Fructose	+
Erythritol	−	Salicin	+	Seminose	−
D-Arabinose	−	Cellobiose	−	Sorbose	−
L-Arabinose	−	Maltose	+	Rhamnose	−
Ribose	w	Lactose	−	Melampyrin	−
D-Xylose	−	Melibiose	-	Increositol	−
L-Xylose	−	Saccharose	+	Mannitol	−
Adonite	−	Trehalose	+	Sorbic alcohol	−
Galactose	−	Inulin	−	α-Methyl-D-mannoside	−
D-Arabinol	−	Melezitose	−	α-Methyl-d-glucoside	−
L-Arabinol	−	Raffinose	−	N-Acetyl-glucosamine	−
Amygdalin	−	Starch	−	2-Keto-gluconate	−
Gluconate	−	Glycogen	+	5-keto-gluconate	−
D-Turanose	−	Xylosic alcohol	−	β-Methyl-D-xyloside	−
D-Tagatose	−	D-Lyxose	−		

Note: + means positive reaction, − means negative reaction, w means weakly positive reaction.

## References

[1] Zakzeski J., Bruijnincx P.C.A., Jongerius A.L., Weckhuysen B.M. (2010). The catalytic valorization of lignin for the production of renewable chemicals. Chem. Rev..

[2] Bandounas L., Wierckx N.J.P., de Winde J.H., Ruijssenaars H.J. (2011). Isolation and characterization of novel bacterial strains exhibiting ligninolytic potential. BMC Biotechnol..

[3] Lee S., Kang M., Bae J.H., Sohn J.H., Sung B.H. (2019). Bacterial Valorization of Lignin: Strains, Enzymes, Conversion Pathways, Biosensors, and Perspectives. Front. Bioeng. Biotechnol..

[4] Masai E., Ichimura A., Sato Y., Miyauchi K., Katayama Y., Fukuda M. (2003). Roles of the enantioselective glutathione S-transferases in cleavage of β-aryl ether. J. Bacteriol..

[5] Santos A., Mendes S., Brissos V., Martins L.O. (2014). New dye-decolorizing peroxidases from Bacillus subtilis and Pseudomonas putida MET94: Towards biotechnological applications. Appl. Microbiol. Biotechnol..

[6] Ahmad M., Roberts J.N., Hardiman E.M., Singh R., Eltis L.D., Bugg T.D.H. (2011). Identification of DypB from rhodococcus jostii RHA1 as a lignin peroxidase. Biochemistry.

[7] Trojanowski J., Haider K., Sundman V. (1977). Decomposition of c-14-labeled lignin and phenols by a nocardia-sp. Arch. Microbiol..

[8] Sahm L.E., Sahm H. (1980). Degradation of Coniferyl Alcohol and Other Lignin-Related Aromatic Compounds by Nocardia sp. DSM 1069. Arch. Microbiol..

[9] Min K., Gong G., Woo H.M., Kim Y., Um Y. (2015). A dye-decolorizing peroxidase from Bacillus subtilis exhibiting substrate-dependent optimum temperature for dyes and b-ether lignin dimer. Sci. Rep..

[10] Chandra R., Chowdhary P. (2015). Properties of bacterial laccases and their application in bioremediation of industrial wastes. Environ. Sci. Process. Impacts.

[11] De Gonzalo G., Colpa D.I., Habib M.H.M., Fraaije M.W. (2016). Bacterial enzymes involved in lignin degradation. J. Biotechnol..

[12] Brown M.E., Chang M.C.Y. (2014). Exploring bacterial lignin degradation. Curr. Opin. Chem. Biol..

[13] Bianchetti C.M., Harmann C.H., Takasuka T.E., Hura G.L., Dyer K., Fox B.G. (2013). Fusion of dioxygenase and lignin-binding domains in a novel secreted enzyme from cellulolytic streptomyces sp. SIRexaa-e. J. Biol. Chem..

[14] Perestelo F., Falcon M.A., De La Fuente G. (1989). Production of vanillic acid from vanillin by resting cells of Serratia marcescens. Appl. Environ. Microbiol..

[15] Allocati N., Federici L., Masulli M., Di Ilio C. (2009). Glutathione transferases in bacteria. FEBS J..

[16] Reiter J., Strittmatter H., Wiemann L.O., Schieder D., Sieber V. (2013). Enzymatic cleavage of lignin β-O-4 aryl ether bonds via net internal hydrogen transfer. Green Chem..

[17] Masai E., Nishikawa S., Morohoshi N., Haraguchi T. (1989). Detection and localization of a new enzyme catalyzing the fl-aryl ether cleavage in the soil bacterium (Pseudomonas paucimobilis SYK-6). FEBS Lett..

[18] Picart P., Sevenich M., Domínguez De María P., Schallmey A. (2015). Exploring glutathione lyases as biocatalysts: Paving the way for enzymatic lignin depolymerization and future stereoselective applications. Green Chem..

[19] Rashid G.M.M., Taylor C.R., Liu Y., Zhang X., Rea D., Fü V., Bugg T.D.H. (2015). Identification of Manganese Superoxide Dismutase from Sphingobacterium sp. T2 as a Novel Bacterial Enzyme for Lignin Oxidation. ACS Chem. Biol..

[20] Koren S., Walenz B.P., Berlin K., Miller J.R., Bergman N.H., Phillippy A.M. (2017). Canu: Scalable and accurate long-read assembly via adaptive k-mer weighting and repeat separation. Genome Res..

[21] Arimoto M., Yamagishi K., Wang J., Tanaka K., Miyoshi T., Kamei I., Kondo R., Mori T., Kawagishi H., Hirai H. (2015). Molecular breeding of lignin-degrading brown-rot fungus Gloeophyllum trabeum by homologous expression of laccase gene. AMB Express.

[22] Xu Q., Zhong H., Zhou J., Wu Y., Ma Z., Yang L., Wang Z., Ling C., Li X. (2021). Lignin degradation by water buffalo. Trop. Anim. Health Prod..

[23] Terramoccia S., Bartocci S., Amici A., Martillotti F. (2000). Protein and protein-free dry matter rumen degradability in buffalo, cattle and sheep fed diets with different forage to concentrate ratios. Livest. Prod. Sci..

[24] Chanthakhoun V., Wanapat M., Kongmun P., Cherdthong A. (2012). Comparison of ruminal fermentation characteristics and microbial population in swamp buffalo and cattle. Livest. Sci..

[25] Wang Z., Wu W., Cui L., Li X., Kulyar M.F.E.A., Xiong H., Zhou N., Yin H., Li J., Li X. (2021). Isolation, characterization, and interaction of lignin-degrading bacteria from rumen of buffalo (Bubalus bubalis). J. Basic Microbiol..

[26] Zhong H., Zhou J., Abdelrahman M., Xu H., Wu Z., Cui L. (2021). The Effect of Lignin Composition on Ruminal Fiber Fractions Degradation from Different Roughage Sources in Water Buffalo ( Bubalus bubalis ). Agriculture.

[27] Garrity G.M., Bell J.A., Lilburn T.G. (2004). Taxonomic Outline of the Prokaryotes Bergey’s Manual of Systematic Bacteriology.

[28] Patel R.K., Jain M. (2012). NGS QC toolkit: A toolkit for quality control of next generation sequencing data. PLoS ONE.

[29] Schubert M., Lindgreen S., Orlando L. (2016). AdapterRemoval v2: Rapid adapter trimming, identification, and read merging. BMC Res. Notes.

[30] Luo R., Lam T.-W., Liu B., Xie Y., Li Z., Huang W., Yuan J., He G., Chen Y., Pan Q. (2012). SOAPdenovo2: An empirically improved memory-efficient short-read de novo assembler. Gigascience.

[31] Chin C.S., Peluso P., Sedlazeck F.J., Nattestad M., Concepcion G.T., Clum A., Dunn C., O’Malley R., Figueroa-Balderas R., Morales-Cruz A. (2016). Phased diploid genome assembly with single-molecule real-time sequencing. Nat. Methods.

[32] Walker B.J., Abeel T., Shea T., Priest M., Abouelliel A., Sakthikumar S., Cuomo C.A., Zeng Q., Wortman J., Young S.K. (2014). Pilon: An integrated tool for comprehensive microbial variant detection and genome assembly improvement. PLoS ONE.

[33] Besemer J., Lomsadze A., Borodovsky M. (2001). GeneMarkS: A self-training method for prediction of gene starts in microbial genomes. Implications for finding sequence motifs in regulatory regions. Nucleic Acids Res..

[34] Huerta-Cepas J., Forslund K., Coelho L.P., Szklarczyk D., Jensen L.J., Von Mering C., Bork P. (2017). Fast genome-wide functional annotation through orthology assignment by eggNOG-mapper. Mol. Biol. Evol..

[35] Moriya Y., Itoh M., Okuda S., Yoshizawa A.C., Kanehisa M. (2007). KAAS: An automatic genome annotation and pathway reconstruction server. Nucleic Acids Res..

[36] Lombard V., Golaconda Ramulu H., Drula E., Coutinho P.M., Henrissat B. (2014). The carbohydrate-active enzymes database (CAZy) in 2013. Nucleic Acids Res..

[37] Raj A., Reddy M.M.K., Chandra R., Purohit H.J., Kapley A. (2007). Biodegradation of kraft-lignin by Bacillus sp. isolated from sludge of pulp and paper mill. Biodegradation.

[38] Wanapat M., Rowlinson P. (2007). Nutrition and feeding of swamp buffalo: Feed resources and rumen approach. Ital. J. Anim. Sci..

[39] Giardina P., Faraco V., Pezzella C., Piscitelli A., Vanhulle S., Sannia G. (2010). Laccases: A never-ending story. Cell. Mol. Life Sci..

[40] Givaudan A., Effosse A., Faure D., Potier P., Bouillant M.L., Bally R. (1993). Polyphenol oxidase in Azospirillum lipoferum isolated from rice rhizosphere: Evidence for laccase activity in non-motile strains of Azospirillum lipoferum. FEMS Microbiol. Lett..

[41] Alexandre G., Zhulin I.B. (2000). Laccases are widespread in bacteria. Trends Biotechnol..

[42] Santhanam N., Vivanco J.M., Decker S.R., Reardon K.F. (2011). Expression of industrially relevant laccases: Prokaryotic style. Trends Biotechnol..

[43] Martins L.O., Durão P., Brissos V., Lindley P.F. (2015). Laccases of prokaryotic origin: Enzymes at the interface of protein science and protein technology. Cell. Mol. Life Sci..

[44] Xu R., Zhang K., Liu P., Han H., Zhao S., Kakade A., Khan A., Du D., Li X. (2018). Lignin depolymerization and utilization by bacteria. Bioresour. Technol..

[45] Ruijssenaars H.J., Hartmans S. (2004). A cloned Bacillus halodurans multicopper oxidase exhibiting alkaline laccase activity. Appl. Microbiol. Biotechnol..

[46] Masai E., Katayama Y., Fukuda M. (2007). Genetic and biochemical investigations on bacterial catabolic pathways for lignin-derived aromatic compounds. Biosci. Biotechnol. Biochem..

